# Anal sexual experience and HIV risk awareness among female sex workers in Dire Dawa, eastern Ethiopia

**DOI:** 10.1186/s41256-017-0047-6

**Published:** 2017-09-08

**Authors:** Yohannes Teka Mazeingia, Lemessa Olijjira, Yadeta Dessie

**Affiliations:** 1 Debre Markos University, Debre Marqos, Ethiopia; 2 Haramaya University, Harar, Ethiopia

**Keywords:** Female sex workers, HIV, Anal sex, Risk awareness

## Abstract

**Background:**

Female sex workers have been disproportionately affected with HIV and anal sexual experience elevate their vulnerability. Anal intercourse has more risk of HIV transmission than vaginal intercourse for receptors that coupled with low condom and proper lubricant use behavior during anal sex. Besides majority of them did not understand HIV transmission risk of anal intercourse. In Ethiopia, studies on anal sexual experience is almost none existent, so the purpose of this study is to explored anal sexual experience and HIV transmission risk awareness among female sex worker in Dire Dawa, Eastern Ethiopia.

**Method:**

Qualitative study with thematic analysis approach was conducted among 18 female sex workers and recruitment of study participants performed until saturation of information. The principal investigator conducted in-depth interviews using local language (Amharic) and it was recorded on audio recorder. Tape recorded data was transcribed and translated to English and entered into open code version 3.4 for coding and theme identification. Data collection conducted simultaneously with data analysis.

**Result:**

Female sex workers practiced anal sex for different themes like financial influence, coercion, intentionally, peer pressure and as a sign of intimacy and love. Coercion, negative attitudes, poor awareness about HIV transmission risks of anal sex and protection capacity of condom and proper lubricants are the identified themes for not using condom and proper lubricants during anal sex by female sex workers. Inaccessibility and unavailability of health services for issues related to anal sex was the core reason for female sex workers’ misperception and risk anal sexual experience.

**Conclusion:**

Female sex workers practiced anal sex without risk reduction approaches and they did not understand exacerbated risk of anal sex to HIV transmission. Stakeholders including ministry of health need to incorporate potential awareness raising tasks and programs about risk of anal sex and methods of risk reduction for female sex workers.

**Electronic supplementary material:**

The online version of this article (doi:10.1186/s41256-017-0047-6) contains supplementary material, which is available to authorized users.

## Background

Since late twentieth century, HIV has become a major global public health problem particularly in Sub Saharan Africa including Ethiopia [1]. HIV hurt female sex works (FSWs) disproportionately than the general adult population (23% Vs 1.2%) [2, 3]. Beside high prevalence of HIV among FSWs, they serve as key transmitter of HIV [4]. Inconsistent condom use, anal sexual experience and multiple sexual partner attributed for FSWs’ vulnerability to HIV and their infectiousness [5]. Anal sex became common sexual experience among commercial sex workers, 13.1% of FSWs in Ethiopia reported as ever-practiced anal sex [6–8].

HIV transmission risk on per act unprotected receptive anal intercourse is 20 times higher than receptive vaginal intercourse [9]. Besides, risk of HIV acquisition is much higher among receptors than inserter that make FSWs extremely vulnerable to HIV than their clients [10]. Significant proportion of new HIV infection can be halted by averting anal sexual behavior of FSWs alone and by enabling them to engage in vaginal sex [11, 12]. Though HIV transmission risk worsen during anal sex, many FSWs did not use condom consistently and only few used proper lubricants during anal sex [7, 13]. In Ethiopia, about 53.1% of FSWs who experienced anal sex did not use condom during anal intercourse [8].

Studies conducted in previous years among FSWs in Ethiopia, focused on exploring and determining risk sexual behaviors giving emphasis to vaginal sex [8, 14–16]. Moreover, most of these studies did not assess FSWs anal sexual experience like how and why they practiced anal sex; and how they understood its risk for HIV transmission [8]. Though studies conducted in other countries discussed anal sexual behavior of female sex workers and their awareness about its HIV transmission risk, exploring the local settings is crucial to design and implement effective HIV prevention programs.

HIV intervention programs in Ethiopia did not give due emphasis to anal sexual behavior of female sex workers and related risk reduction approaches [2]. The finding of the study will be useful to influence decision makers to incorporate intervention programs and tasks to halt new HIV infection attributed to female sex workers anal sexual experience and to reach the goal of ending HIV/AIDS by 2030. Therefore, the purpose this study is to explore anal sexual experience of female sex workers; and how they understand risk of HIV transmission through anal sex.

## Method

Qualitative study was conducted in Dire Dawa town where HIV prevalence was higher than national level, 4% Vs 1.5% [16]. The principal investigator executed in-depth interview among 18 female sex workers who experienced anal sex in commercial sex work. The study was undertaken from February 25 through May 24, 2016. Study participant female sex workers were screened based on self-reporting of penile anal sexual experience. Number of study participants was determined with saturation of information and we recruited diverse female sex workers based their age, sex work type, experience as peer educator and motherhood.

The first three study participants or seeds were recruited through agents. Then each study participant recruited one or two other FSWs who have anal sexual experience. During the recruitment process, first the recruiter individual would tell new study participants about the study and as the principal investigator would meet them for further information. Then if they became voluntary to participate, the recruiter person would give recruited women’s phone number to the principal investigator. Then the principal investigator met each recruited participant in place, they would be accessed easily and can ensure privacy to talk. Study participants were informed about the purpose of the study and confidentiality of the information they provided. We took informed written consent from each study participant prior to each interview. Each study participant was paid about 13$ USD for compensating the business they loss while they participate in the study which is estimated based on the minimum amount of money they were paid for per act vaginal intercourse, which was at least two times lower than that clients paid for per act anal sex. Besides, we provided extra 4$ USD to recruiter individuals for successfully recruiting one eligible FSW to the study.

Then the principal investigator collected data with in depth interviews which, were conducted with local language (Amharic) using semi structured interview guide. Each interview took 1 hour to one and half hour. To increase trustworthiness the principal investigator approached them friendly; moreover we provided brief description about the study for each study participant to enable them speak freely and show interest on the research before conducting the interview. Interviews were performed in place and time that agreed by both parties to ensure safety and privacy for study participants. During interviews, we forwarded crosschecking questions to them to be sure with their responses and consistency of their ideas.

The following are some of the operational definitions used in this study.

Anal Intercourse/sex: penetrative anal sex by a male sexual partners’ penis or putting his penis in female sex workers anus [17].

Female sex workers (FSWs): is female who had sex for exchange of money in cash [18].

Risk awareness: understand true risk of HIV transmission through anal intercourse or be aware as anal intercourse carry exacerbated risk for HIV transmission than vaginal intercourse [19].

Data analysis was performed with thematic analysis approach and it was executed concurrently with data collection. At the end of each interview we listened the tape recorded interviews repeatedly and then it was transcribed. Then after, we removed jargon data and translated the transcribed data to English. The principal investigator read the translated data repeatedly and transferred in to Open Code software version 3.4., for easy of coding and theme identification. Open coding and themes identification was performed inductively. Then major themes were identified from the existing sub-themes and we used major themes for the final analysis. In subsequent interviews, codes and themes were modified based on the codes and themes from prior interviews or new codes and themes were emerged from the data.

## Result and discussion

### Result

Study participants had mean age of 24.8 years, ranging from 18 to 39 years. Except one, all study participants had formal education. About ten of 18 FSWs had at least one child. Four of them were peer educators and two women identified themselves, as they were diagnosed positive for HIV. Moreover about ten and five FSWs were venue based and phone based commercial sex workers respectively. Duration of their stay in commercial sex work varies from one to 10 year. (Table 1 on the annex).

**Table 1:** socio demographic and other characteristics of study participant FSWs, 2016

Code of the women	Age in yrs	Marital status	# of Child(children)	Education in grade	Place of birth	Extra job	Years in sex work
A	25	Divorced	1	10^+1^	Dire Dawa	Waiter	4
B	21	Divorced	0	4	Dire Dawa	No	6
C	25	Divorced	1	9	Dire Dawa	Pimp	6
D	28	Widowed	1	3	Dire Dawa	Waiter	2
E	22	Divorced	1	10	Dire Dawa	Waiter	2
F	30	Widowed	1	6	Dire Dawa	Waiter	6
G	28	Widowed	2	9	Dessie (rural)	Waiter	2
H	19	Single	0	9	Dire Dawa	No	2
I	19	Divorced	0	9	Addis Ababa	No	1
J	18	Widowed	0	10	Dire Dawa	No	4
K	25	Divorced	0	9	Dire Dawa	Waiter	4
L	30	partner	0	Illiterate	Ambo	No	10
M*	24	Divorced	3	9	Jigjiga	Waiter	5
N*	27	Partner	1	8	Harar	Peer educator	10
O	23	Partner	0	8	Dire Dawa	Peer educator	4
P	18	Partner	0	8	Dire Dawa	No	4
Q	39	Divorced	2	6	Dire Dawa	No	1
R	26	Divorced	1	7	Bedelo (rural)	Peer educator	6

*HIV positve

### Why women practiced anal sex in commercial sex work?

Female sex workers experienced anal intercourse for different reasons and five major themes were identified from these reasons.

Financial influence: FSWs practiced anal sex for two types of financial influences. Primarily relatively high payment for anal sex than vaginal sex influenced them to accept clients’ (Additional file 1) offers for anal sex. The other perspective of financial influence raise from their poor economic power and from their immediate demand of money, which forced them to accept any offers from clients including for anal sex whether they will be paid well or equivalent with the money they could make from vaginal sex.

*…the thing that you should know is; if they paid me well, for what would I be concerned? Why would I deny their offer? I am not selling myself for money but it is part of this work… I was just shocked when I heard the amount of money the man offered me for the first time. Any girl would faint if she heard as she would be paid about 3000 or 4000 birr for a night stand anal sex. No one would be able to reject such amount of birr… J (phone based FSW*

*…The only reason I accepted offers for anal intercourse from men was that I should do it and get money to survive. When I engaged in anal intercourse for the first time, I had slept for days... it was a matter of life or survival to engage in anal intercourse. If I did not accept those offers, what other options do I have? I have no other means of income other than sex work; moreover, I had no family to live with. If I did not do sex work business today, tomorrow I will have nothing to pay for something I need to buy like food, drink and so on. Since I will have nothing to eat and drink tomorrow, I need to go to sex working site for doing business and sleep with men for money. Once I went for sex work, I should accept offers for anal intercourse too… k (Venue based FSW)*

Coerced sex and poor decision-making power: clients engaged in anal sex with FSWs forcefully without the will of FSWs. Different situations increased FSWs vulnerability for experiencing coerced anal sex like being drank of FSWs and unsafe hotel or gust houses.

*…I would not engage in anal intercourse whatever money he paid me. You know why, because the money he gave me might not even cover medical bills for problems happened on me due to my anal sexual experience… I just engaged in anal intercourse once while I was very drunk. Did you hear me? After that moment, I did not want to try it again. That day, I only remembered the sex we did once and as I slept after that, since I was very drunk. Then I shouted and disturbed the surrounding due to the pain I felt when he inserted his penis in my anus when I was in deep sleep… There are small hotels here in Dire Dawa where nobody came for help when we (FSWs) shout, I hate spending a minute in these hotels. I feel excited when clients took me to standard hotels, because standard hotels always registered full identification information of their gusts. So these men will go nowhere hurting me… R (venue based FSW)*

Intentionally: they practiced anal intercourse to enjoy sexual pleasure particularly among FSWs who developed extreme desire for anal sex due to their repeated anal sexual experience. Besides these women did so to get relief from unpleasant sensation they experienced when they avoided anal sex. Female sex workers also practiced anal sex once they adapt it considering it as part of commercial sex work, though it was not pleasurable for them.

*…nothing influenced me to practice sexual intercourse from behind; I did it on my will. Even sometimes, I feel ashamed to ask men to have sex with me from behind, when I want to satisfy my sexual desire. I need men to practice sex with me from behind, though I do not request them to do so since I feel ashamed for developing desire for anal intercourse. I never asked men to do so but if any client requested me for anal intercourse, I will accept his offer with pleasure… H (phone based FSW)*

*…Variety of men, like their skin color, came to us sex workers to engage in sexual intercourse, so there would be some men who wanted to practice anal sex. When they request me for anal sex, I will accept their offer since it is my job to satisfy my clients… Q (venue based FSW)*

As sign of intimacy and love: the fourth major theme that FSWs practiced anal sex was to express their intimacy and love to men. They experienced anal sex with specific regular clients and partners for these reasons.

*...When my spouse came home looking tired, he always wanted to have anal intercourse and I did not deny him from doing so since he was my real husband. I should practice sexual intercourse with my husband in a way he want to do with me... F (venue based FSW)*

*…for me, I have about seven regular clients to practice anal sex alone. They have the power to pay whatever I ask them. Mostly I do not engage in anal intercourse with other customers. Actually, I do not like practicing anal intercourse with other men other than those seven men whom I like very much … N (HIV Positive Street based FSW)*

Peer influence: female sex workers who never experienced anal intercourse mostly influenced by their peers who have extensive anal sexual experience particularly when they try and practice anal intercourse for the first time.

*....It was not new for my friends to practice anal intercourse. One of my friends told me as one of the man would pay me well if I will go with him and practice anal intercourse. She influenced me to try it with him and I did it because of her... E (venue based FSW)*

### Condom use practices and their awareness of HIV transmission risk through anal sex

Nine of the 18 FSWs use condom inconsistently during anal intercourse and three themes emerged to explain their inconsistent condom use behavior. The first theme is poor decision-making power of women and they practiced unprotected anal sex due to objection of their sexual partners for not using condom. These objections from FSWs’ sexual partners vary from coercion and rape to influencing FSWs to accept their offer for unprotected anal intercourse by threatening them, as they would not pay for the vaginal sexual intercourse they already practiced.

*.... He just inserted his penis through my anus when I was in state of deep sleep... Nooooo he did not use, he did not use any condom ... it is mandatory to use condom ... Some standard hotels did not put condom at bedrooms. Actually, I always have condom with me... R (venue based FSW)*

*...mostly men did not want to use condom during anal intercourse. They thought anus would be relaxed and would loss its tightness if they used condom, so they prefer unprotected anal sex since they do not like anus to lose its tightness. Moreover, mostly they do not want lubricates applied on condom... When I told them, as we should protect ourselves from different problems by using condom, you know what they would say, ‘a real sex is when you do it freely and without condom’.... A (venue based FSW)*

Pleasure principle: Female sex workers practiced unprotected anal intercourse to enjoy maximum pleasure from their sexual engagement. This because they develop negative attitude towards condom and perceive as condom will decrease their pleasure during sexual intercourse.

*…Whenever I agree with a client for anal intercourse, primarily I ask them how they will practice it and whether they will ejaculate out of my anus or not. Some clients want to ejaculate inside my anus. If the client agree not to ejaculate inside me, I will offer him what he want. You know why I suggest them to ejaculate outside my anus it is because I dislike using condom since condom causes dryness during intercourse. If you ever tried condom, the lubricant it has would fad up as the intercourse prolonged. Therefore, condom do not give me comfort during intercourse. If they do not ejaculate inside me, I do not care whether they use condom or not or whoever the client is. You know why I dislike condom, the lubricant condom has, has very disgusting smell and I hate it. I did not use condom mostly… E (venue based FSW)*

Misperceptions about condom: female sex workers believe using condom during anal sexual intercourse is not necessary and they did not use it. They thought so in two scenarios in which either they do not aware the risk of HIV transmission through anal intercourse or since they believe as they are no more at risk of HIV though they practiced unprotected anal sexual intercourse.

*… My clients tell to me, as anal intercourse do not transmit any disease including HIV. Mostly I do not use condom during anal intercourse since I know, as there is no risk of HIV from practicing unprotected anal intercourse … C (venue based FSW)*

*… I only fear not to be pregnant whenever I went with a man to practice sexual intercourse. I do not fear other issues; even I do not care about HIV since I am already infected with HIV. I am taking anti retro viral drugs currently… N (HIV positive peer educator, street based FSW)*

None of the study participants used proper lubricants though about 11 of them used petroleum-based lubricants like Vaseline consistently. About three themes are identified to explain why FSWs did not use compatible lubricants during anal intercourse.

Lack of awareness about lubricants: it is the first theme that enable FSWs to practice anal sex without proper lubricant. They did not use proper lubricants since they did not aware its advantage for minimizing HIV transmission risk during anal intercourse and the proper type of lubricants. Petroleum-based lubricants are the commonly used lubricants for anal intercourse by FSWs and this is because they misunderstand petroleum based lubricants as proper type of lubricant and they do not aware their adverse effects. Moreover, they also perceive as condom has adequate lubricant for practicing anal sex and misperceived as additional lubricant is not necessary if they practice anal sex with condom. Lack of awareness about proper lubricant use during anal sex among FSWs attributed to poor health information dissemination tasks from health professionals on issues related to anal intercourse. Besides FSWs themselves did not use opportunities to discuss with health service providers about proper lubricant use when they attended health facilities for other services.

*…condom itself has lubricant and I think using condom is enough. Since I use condom always, I never thought to use additional lubricant … D (venue based FSW)*

*… We use Vaseline adding it on the condom, because condom will lose its lubricating capacity gradually during intercourse. The anal mucosa is sensitive and easily traumatized, the Vaseline that will be added on the condom will lubricate anus. Mostly, I use Vaseline as lubricate…I never thought about side effects from using Vaseline… B (phone based FSW)*

Negative attitude about lubricants: Some of FSWs are not interested to use any type of lubricant though they aware the advantage of using additional lubricants with condom. The negative attitude of FSWs for lubricant raise from their previous experiences, fear of side effects and misperceptions.

*...I heard from my friends, as there is lubricate that can be used during anal intercourse... Nevertheless, I did not use it... Even the lubricant of condom is difficult for my vagina, give alone using lubricants that I did not know well... G (venue based FSW)*

Coerced anal sex: female sex workers practiced anal intercourse without proper lubricants in times they experienced anal intercourse due to rape. They were not able to use lubricants in such scenarios since they were not able to deal with their sexual partner and convince their sexual partners to use proper lubricants.

*... I never use any thing as lubricant. Sometimes men might lubricate their penis with saliva and before inserting it on me. Men know as, anal sex is painful since they can observe my suffering on my face while we practice it. If the man understand my suffering and believe as I have no power to oppose him from practicing anal intercourse, at least he will lubricate his penis with saliva... A (venue based FSW)*

Female sex workers understand risk of HIV transmission through anal intercourse in three schemes. The first group of FSWs perceived anal sex as HIV risk free sexual behavior. They believe as, there should have contact between sperm and vaginal cavity to transmit HIV during sexual intercourse.

*...how HIV could be transmitted through anus? How anus will have contact with vagina and uterus and transmit HIV; because anus is located on behind in opposite to vagina and uterus, which are located on the front. I think HIV does not transmit through eating together and practicing anal intercourse with HIV positive person, the two have no difference... F (venue based FSW)*

The second group of FSWs perceived as there is risk of HIV transmission through anal intercourse but they do not aware its exacerbated risk for HIV transmission. They perceive risk of HIV transmission during anal intercourse is either minimal or comparable with HIV transmission risk through vaginal intercourse.

*... Many individuals thought as HIV transmit only through normal (vaginal) sex, the route that God allow for us to practice sexual intercourse. However, to my level of understanding, anus is also part of my body so it can transmit HIV like the one it will be transmitted through vaginal intercourse... I think both vagina and anus had equal chance for transmitting HIV... I (phone based FSW)*

The third group of FSWs aware about the elevated risk of HIV transmission during anal sex. They thought the exacerbated risk of HIV transmission during anal intercourse is attributed to high vulnerability of anus for friction and trauma due to thin layer and tight nature of anus. Beside this fragility of anal mucosa during intercourse precipitated with lack of anal secretion.

*... HIV can be transmitted through anal intercourse more than it can be transmitted through vaginal intercourse. There is more friction and more pain during anal intercourse than during vaginal sex since anus is very fragile and has a very thin wall. I think anal mucosa is the one that can be traumatized and ulcerated easily than vaginal mucosa. When we see vagina it produce its own secretion during intercourse. There is no secretion from anus during anal intercourse... A (venue based FSW)*

### Discussion

Financial influences were one of the major themes that FSWs engaged in anal sex in this study. These financial influences raise from either financial problem or high payment for anal intercourse. Provision of extra money was a common reason for numerous FSWs to practice anal intercourse in other studies too [13, 20]. Financial problems influenced FSWs to practice anal sex since many women entered in to commercial sex work to make money and they got more money when they practiced anal intercourse than vaginal intercourse. Moreover, they engaged in anal intercourse to get relief from the financial trouble they faced which were provoked with lack of extra means of income in addition to commercial sex work [21] and having family to support [22]. Particularly, if FSWs have children, about half of the study participants have at list one child; they will take risks to give care for their kids [21].

Coercion and limited decision-making power of female sex workers to forbid clients from practicing anal intercourse was the other theme that FSWs practiced anal intercourse. Clients forcefully practiced anal sex with FSWs in other studies too [13, 17, 23]. They became vulnerable to coerced anal intercourse if they are drunk [24], because alcohol diminished their decision-making power and weaken their ability to control their environment while they were with clients [25, 26]. Besides, unsafe working environment around hotels or gust houses including lack of support from hotel guards increased FSWs susceptibility for coercion.

When clients forced FSWs to practice anal intercourse, they did not use risk minimization methods like condom and proper lubricants. Other studies discussed that FSWs were less likely to use condom if they practiced anal intercourse forcefully [7]. Even in times when they practiced anal intercourse with their will, clients might refuse to use condom and would force FSWs to practice unprotected anal sex [27]. Clients influenced FSWs to engage in unprotected anal sex by offering extra money [28]. Moreover, many FSWs lack negotiation skills to convince clients on condom use [29].

Half (9/18) of study participants did not use condom consistently during anal intercourse. This finding is consistent with study conducted in North West Ethiopia and in India, which discussed as only around half of FSWs were using condom during anal intercourse [8, 30]. Nevertheless almost all of FSWs were using condom during vaginal intercourse in 2013 national MARP survey in Ethiopia [2]. This difference in condom use behavior during anal sex and vaginal sex is due to lack efforts from stakeholders to increase condom use practice during anal intercourse as compared to their efforts on improving female sex workers condom use behavior during vaginal intercourse.

Negative attitudes towards condom and misperceptions on importance of condom during anal intercourse were discussed by FSWs for practicing unprotected anal intercourse. They misperceived significance of using condom during anal intercourse due to lack of awareness about risk of anal intercourse for HIV transmission. This finding is supported by study conducted in Kenya where female sex workers practiced unprotected anal sex due to their negative attitude towards condom [7]. Poor health service consumption of Female sex workers for concerns related to anal intercourse exacerbate their negative attitudes for condom. Moreover, anal sexual behavior does not get due emphasis under national HIV prevention programs and it is not recognized as route for sexual transmission of HIV in the country. Stakeholders do not undertake tasks to minimize HIV transmission risk through anal intercourse [2]. Besides, health service providers also do not provide health information about condom use behaviors and risk of anal sex for HIV transmission. Even clinics prepared to provide services for FSWs alone did not offer the least, counseling service about risk of HIV transmission during anal sex when they attended at the clinic for consuming other available health services.

Similarly, none of the study participants used proper lubricants during anal sex and Vaseline was the commonly used lubricant. Lack of awareness about lubricants, negative attitude for lubricants and misperception as condom is sufficient protection measure during anal intercourse are the major themes for not using proper lubricants by female sex workers during anal sex. They lack awareness on the proper types of lubricant to be used during anal sexual practice and adverse effects of using petroleum based lubricants.

These themes are routed from not understanding the role of proper lubricant in minimizing the elevated HIV transmission risk during anal intercourse. Poor knowledge of FSWs about advantage of proper lubricants for anal sex precipitated with their lack of health services including information dissemination about proper lubricants. Give alone health services provided at the grass root level of the health system, distribution of proper lubricants is not intended by government in its 5 year national HIV/AIDS prevention strategic plan from 2015 to 2020 [2]. Though other literatures on hand did not discuss why FSWs did not use proper lubricants during anal sex, studies conducted in Kenya, India and South African countries reported as FSWs either did not use lubricant at all or used petroleum based lubricants during anal intercourse [7, 13, 31].

Female sex workers inadequately understand HIV transmission risk of anal intercourse. Majority (13/18) study participants do not understand its exacerbated risk for HIV transmission and most of them (9/13) did not aware as HIV can be transmitted through anal intercourse. The worst scenario was when peer educators and HIV positive FSWs who are under HIV care and treatment did not understand risk of HIV transmission through anal intercourse. They do not attend health services for issues related to anal intercourse. Beside, health professionals do not address these issues during their discussion with FSWs about HIV. Therefore, FSWs perception on anal sex related issues including on risk minimization approaches are mannered on their experience and the information they got from their clients and peers.

Misunderstanding risk of HIV transmission through anal intercourse among FSWs is commonly discussed in other studies as well. Female sex workers discussed about non-HIV related risks of anal intercourse rather than risk of HIV transmission when they were asked about possible risks of anal intercourse in studies conducted in Uganda and India [20, 32]. Moreover, only around quarter of FSWs reported as HIV can be transmitted through AI in India [23]. The study conducted in North West Ethiopia among FSWs also discussed that FSWs did not aware HIV transmission risk of anal intercourse [8]. It was also reported as women did not understand how they would be at risk for HIV infection when they practiced anal intercourse in study conducted in USA [17].

The study has certain limitations. Though we tried to increase its trustworthiness through different approaches, we did not used participant checking to get confirmation from participants on the analysis of the data collected from them. Since anal sex is not a socially accepted behavior, there might be social desirability bias on the information gained from study participants. Therefore readers need to consider these things while they use this article.

## Conclusion

Female sex workers practice risk anal sex for different reasons without protective measures and they do not understand its exacerbated risk for HIV transmission. This will hinder the countries and global plan on fighting against HIV. So, we recommended Dire Dawa city administration health bureau, Ethiopian federal ministry of health and other stakeholders to consider anal intercourse as one route of HIV transmission and incorporate appropriate intervention programs including behavioral change communication activities for these group of population. Moreover, we recommend further research on estimating contribution of anal intercourse for new HIV infection at national level and innovative approaches of HIV prevention on female sex workers who practice anal intercourse that might inform policy and implementation.

## Additional file

Additional file 1: Clients of FSWs for anal intercourse (DOCX 12 kb)

## Abbreviations

AIDS: Acquired Immune Deficiency Syndrome
FSW: Female Sex Worker
HIV: Human Immunodeficiency Virus
