# Supplementary material for: Anal sexual experience and HIV risk awareness among female sex workers in Dire Dawa, eastern Ethiopia
Source: Glob Health Res Policy. 2017 Sep 8;2:27. doi: 10.1186/s41256-017-0047-6 (PMC5683548; doi:10.1186/s41256-017-0047-6)
Supplement: Additional file 1: — Clients of FSWs for anal intercourse (DOCX 12 kb) [file 41256_2017_47_MOESM1_ESM.docx]

Clients of FSWs for anal intercourse

Female sex workers were requested for AI by many of their clients but these clients varied from sex worker to sex workers. The commonly mentioned clients for AI by FSWs were rich, married, elderly and socially known and respected men. These types of men were commonly discussed by FSWs both for requesting AI and for engaging in it with FSWs.

*...By the way, many men asked me for anal intercourse. It is difficult to mention them. Youths were not asking to have such type of sex. It is more common among old adults or those who are on the way to be elderly and married men to ask for anal intercourse... A (Hotel FSW)*

*...Most of the time they were elderly and socially respected individuals; youths might ask sex in different positions to practice what they saw in pornographic movies, otherwise they would not ask for anal intercourse... H (HIV positive street FSW)*

Financial power of elderly men, inability to engage in AI at marriage and respect for their wives were discussed by FSWS as justification for why it was common among respected, elderly and married men than youths. Therefore, FSWs would be better alternatives to engage in AI.

*... It might be due to that they could not have sex openly with their wife in their marriage life; or they might not want to hurt the mother of their children. But if they thought we sex workers are also their wives, sisters or daughters and we are a female too... C (Hotel FSW)*

*... Only those men who were confident enough on their financial ability would have desire to engage in anal intercourse with sex worker... I (phone based FSW)*

*..... it is because of their age, since they passed through a life without knowing about anal intercourse when they were young; they needed to try it now. If you look at youth men since they already knew about it and they passed through it at their age, it was not their concern to engage in anal intercourse with sex workers... M, HIV positive peer educator (hotel FSW)*

Foreigner men were also reported as client for AI especially by phone based FSWs. although elderly men were mentioned as common clients of FSWs for AI, youths were also mentioned as clients for AI especially among low class FSWs. Moreover FSWs also engaged in AI with their partner or husband too.

*...Most of the men who asked for anal intercourse were foreigners, especially those who came from France and China... P (phone based FSW)*

*...Youths want to learn and try many things and they will do it with sex workers only... Anal intercourse is not only common among the youth men but it is also common among youth female sex workers too... D (Hotel FSW)*

*...it was not my first time to engage in anal intercourse, I started it with my husband who was the father of my girl... F (hotel FSW)*
